# Strong Selective Sweeps on the X Chromosome in the Human-Chimpanzee Ancestor Explain Its Low Divergence

**DOI:** 10.1371/journal.pgen.1005451

**Published:** 2015-08-14

**Authors:** Julien Y. Dutheil, Kasper Munch, Kiwoong Nam, Thomas Mailund, Mikkel H. Schierup

**Affiliations:** 1 Institut des Sciences de l'Évolution–Montpellier (ISEM), UMR 5554, CNRS, Université Montpellier 2, Montpellier, France; 2 Bioinformatics Research Centre, Aarhus University, Aarhus, Denmark; 3 Department of Bioscience, Aarhus University, Aarhus, Denmark; Institute of Science and Technology Austria, AUSTRIA

## Abstract

The human and chimpanzee X chromosomes are less divergent than expected based on autosomal divergence. We study incomplete lineage sorting patterns between humans, chimpanzees and gorillas to show that this low divergence can be entirely explained by megabase-sized regions comprising one-third of the X chromosome, where polymorphism in the human-chimpanzee ancestral species was severely reduced. We show that background selection can explain at most 10% of this reduction of diversity in the ancestor. Instead, we show that several strong selective sweeps in the ancestral species can explain it. We also report evidence of population specific sweeps in extant humans that overlap the regions of low diversity in the ancestral species. These regions further correspond to chromosomal sections shown to be devoid of Neanderthal introgression into modern humans. This suggests that the same X-linked regions that undergo selective sweeps are among the first to form reproductive barriers between diverging species. We hypothesize that meiotic drive is the underlying mechanism causing these two observations.

## Author Summary

Because the speciation events that led to human, chimpanzee and gorilla were close in time, the genetic relationship of these species varies along the genome. While human and chimpanzee are the closest related species, in 15% of the genome, human and gorilla are more closely related, and in another 15% of the genome the chimpanzee and gorilla are more closely related—a phenomenon called incomplete lineage sorting (ILS). The amount and distribution of ILS can be predicted using population genetics theory and is affected by demography and selection in the ancestral populations. It was previously reported that the X chromosome, in contrast to autosomes, has less than the expected level of ILS. Using a full genome alignment of the X chromosome, we show that this low level of ILS affects only one third of the chromosome. Regions with low level of ILS also show reduced diversity in the extant populations of human and great apes and coincide with regions devoid of Neanderthal introgression. We propose that these regions are targets of selection and that they played a role in the formation of reproductive barriers.

## Introduction

Despite constituting only 5–6% of the human genome, the human X chromosome is important for elucidating evolutionary mechanisms. Because of its particular inheritance pattern and its cosegregation with the very different Y chromosome, evolutionary forces may act upon it in different ways than on the autosomes [1,2]. Thus contrasting the evolution of the X chromosome with that of the autosomes provides clues to the relative importance of different evolutionary forces.

Hemizygosity of males implies that there are fewer X chromosomes than autosomes in a population (3/4 for even sex ratios). Thus, genetic drift is expected to be relatively stronger on the X chromosome. New variants with recessive fitness effects will also be selected for or against more efficiently on the X chromosome, where they are always exposed in males, than on the autosomes, potentially overriding the increased genetic drift.

Empirical studies have shown that nucleotide diversity is more reduced around genes on the X chromosome than on the autosomes [3–5]. This has been interpreted as the result of more efficient selection on coding variants on the X chromosome, which affects linked positions around the genes. However, no distinction is made here between linked effects of positive selection (genetic hitchhiking [6]) and linked effect of selection against deleterious mutations (background selection [7]). For recessive variants, hitchhiking is expected to be more wide ranging for X chromosomes, whereas a different distribution of fitness effects of deleterious variants on the X is needed to cause stronger background selection on the X. Contrasting non-synonymous and synonymous substitutions with non-synonymous and synonymous polymorphisms, several recent studies have reported evidence for more positive selection on protein changes on the X chromosome in both primates and rodents [8–11]. Whether this is due to hemizygosity, different gene content of the X chromosome, antagonistic selection between sexes being more prevalent on the X chromosome, or some fourth reason is not known.

A separate observation is that the X chromosome in most investigated species is disproportionately involved with speciation, as it (i) contributes disproportionately to hybrid incompatibility (the large X effect) and (ii) together with the Y chromosome is responsible for stronger hybrid depression in males than in females (Haldane’s rule). We refer to Laurie (1997) [12] and Schilthuizen, Giesbers and Beukeboom (2011) [13] for several non-exclusive hypotheses for the underlying genetic mechanisms leading to Haldane’s rule.

Recent introgression from Neanderthals into modern humans was recently reported to be far less common on the X chromosome than on the autosomes. This can be interpreted as evidence for emerging incompatibilities between the two species preferentially residing on the X chromosome [14]. It has been suggested that incompatibilities can accrue due to genetic conflicts between the X and the Y [15–19] and some hybrid incompatibility factors in Drosophila do show evidence of causing meiotic drive [20].

We, and others, have previously reported that the X chromosome shows much less divergence between humans and chimpanzees than expected from autosomal divergence [21–23]. This observation is not based on the nucleotide divergence of the X chromosome versus the autosomes—which will be affected by a difference in mutation rate—but on estimating the effective population size of the ancestral species from the proportion of discordant gene trees.

Because the speciation event between human and chimpanzee and the speciation event between the human-chimpanzee ancestor and the gorilla occurred close in time, around 30% of the autosomal genome shows a gene tree different from the species tree—a phenomenon called incomplete lineage sorting (ILS). The expected amount of ILS depends on the difference between the two speciation times and the effective population size in the human-chimpanzee ancestor. For estimates of the two speciation times in question [24], and assuming that the effective population size of the X chromosome is three quarters of that of the autosomes, the X chromosome is expected to show 24% of ILS. The observed mean amount of ILS, however, is around 15%.

We recently reported that certain regions of the X chromosome in different great ape species often experience what looks like very strong selective sweeps [18]. Here we study the amount of incomplete lineage sorting between human, chimpanzee and gorilla along the X chromosome. We observe a striking pattern of mega-base sized regions with extremely low amounts of ILS, interspersed with regions with the amount of ILS expected from the effective population size of the X chromosome (that is, three quarters that of the autosomes). We show that the most plausible explanation is several strong selective sweeps in the ancestral species to humans and chimpanzees. The low-ILS regions overlap strongly with regions devoid of Neanderthal ancestry in the human genome, which suggests that selection in these regions may create reproductive barriers. We propose that the underlying mechanism is meiotic drive resulting from genetic conflict between the sex chromosomes, and that this is caused by testis expressed ampliconic genes found only on sex chromosomes and enriched in the regions where we find signatures of selective sweeps.

## Results

### Distribution of incomplete lineage sorting along the X chromosome

**Fig 1:**
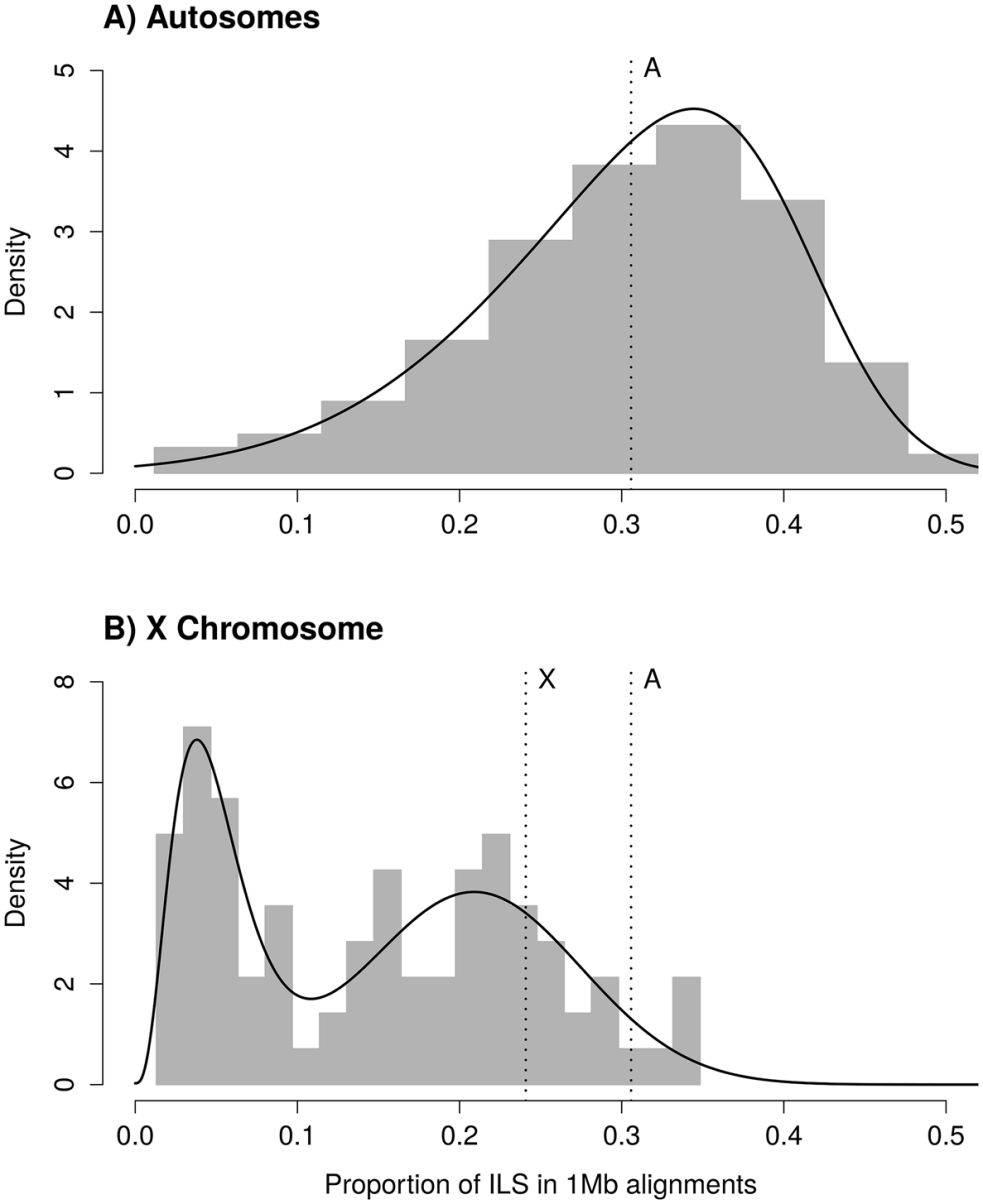
Distribution of incomplete lineage sorting (ILS) along the human genome for autosomes (A) and the X chromosome (B). Grey bars show the distribution of ILS as estimated from the posterior decoding of the CoalHMM model. Solid black lines show the best fit of a skewed normal distribution in (A) and a mixture of a gamma and a Gaussian distribution in (B). The A-labeled vertical line show the median of ILS on the autosomes (A), reported on the X chromosome (B). The X-labeled vertical line shows the expectation of ILS on the X chromosome based on the estimate of ILS on the autosomes (assuming an effective population size three quarters of the effective population size of the autosomes). The second mode of the distribution of ILS on the X chromosome matches this expectation.

To explore the pattern of human-chimpanzee divergence across the full X chromosome we performed a detailed analysis of the aligned genomes of human, chimpanzee, gorilla and orangutan [21]. Using the coalescent hidden Markov model (CoalHMM) approach [25], we fitted a model of speciation by isolation, with constant but distinct ancestral effective population sizes for the human-chimpanzee (HC) and the human-chimpanzee-gorilla (HCG) ancestors. The parameters of the model are (i) two speciation times *τ*_*HC*_ and *τ*_*HCG*_ for human vs. chimpanzee and for HC vs. gorilla, respectively, (ii) two ancestral population sizes *θ*_*HC*_ and *θ*_*HCG*_ for the HC and HCG ancestral populations, respectively, as well as the recombination rate *r* assumed to be constant along both the alignment and phylogeny. An additional parameter is used to account for the divergence with the outgroup sequence. The speciation time, effective population size and recombination rate parameters are scaled according to 2.*Ne*.*u*.*g*, 2.*Ne*.*u* and *u*, respectively, where *u* is the mutation rate per generation, *g* the generation time and *Ne* the population size of a reference extant species [22,25]. Extant population sizes are not parameters of the model, and only serve for the purpose of scaling parameters. To account for putative variation of parameters along the genome alignment, we estimated demographic parameters in non-overlapping 1 Mb windows. We inferred the proportion of ILS using posterior decoding averaged over each of these 1Mb windows. The expected proportion of ILS in a 3-species alignment is given by the formula:

$$Pr\left(ILS\right)=\frac{2}{3}\times exp\left(-\frac{\Delta \tau }{\theta }\right)$$

where *Δτ* is the difference in speciation times and *θ* is the ancestral effective population size of the two most closely related species [26,24] (see also [27]). Estimates of these parameters from the gorilla genome consortium are *Δτ* = 0.002468 and *θ* = 0.003232 [21]. From these parameters, the expected mean proportion of ILS is 31.06%. The observed distribution of ILS proportions on autosomes follows a negatively skewed normal distribution, with a mean of 30.58% (Figs 1A and S1 for individual chromosome distributions).

**Fig 2:**
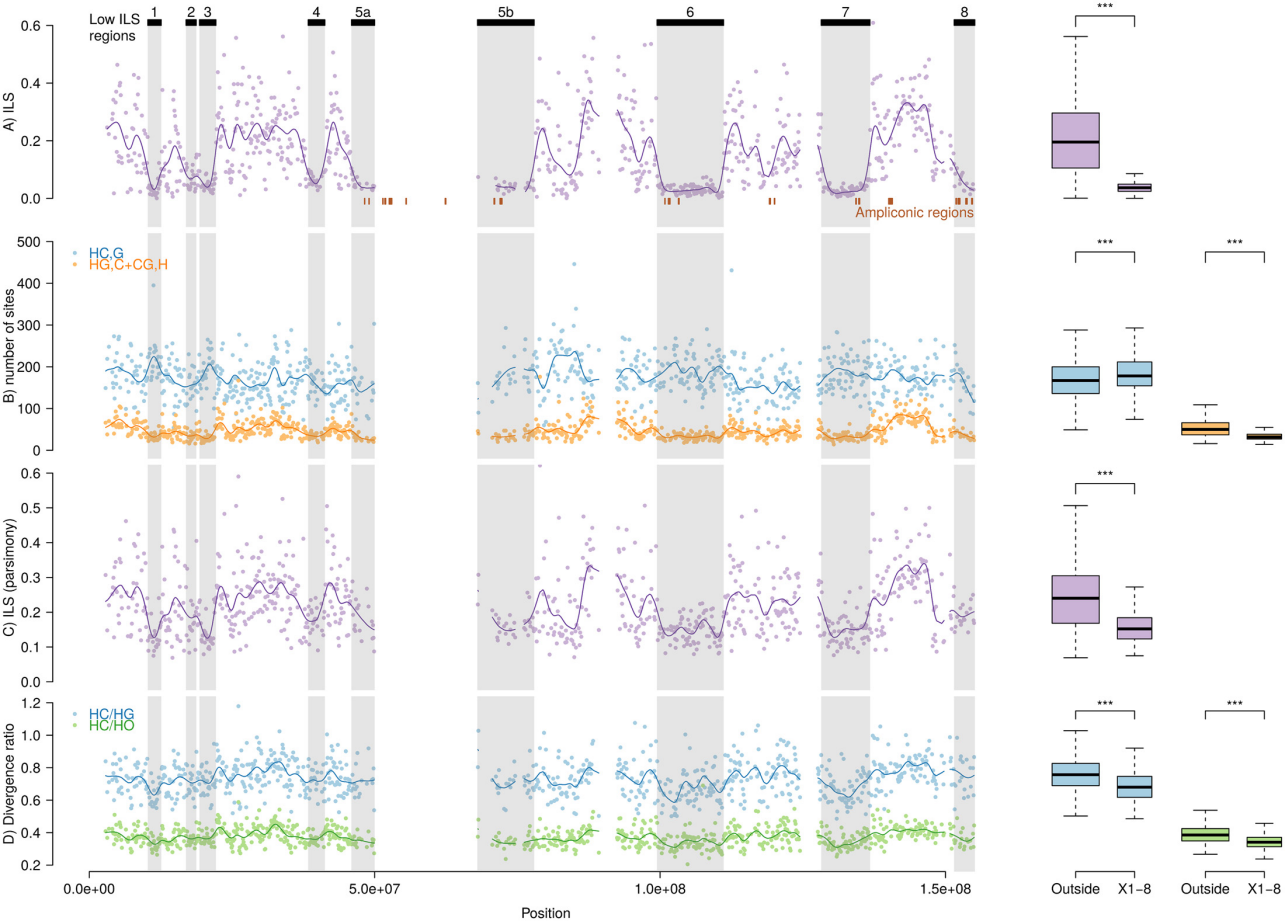
Patterns of incomplete lineage sorting along the X chromosome. Graphs on the left show variation along the chromosome, graphs on the right contrast the distribution of low-ILS regions vs. the rest of the chromosome. Significance codes are according to Wilcoxon rank test. Rows: (A) Proportion of inferred ILS in individual non-overlapping 100 kb windows and a fitted spline. Inferred regions with low ILS are shown on top, and reported on all figures. (B) Frequencies of parsimony informative sites in 100 kb windows, supporting both the canonical genealogy (HC),G and the alternative ones (HG),C and (CG),H together. (C) ILS as estimated by the proportion of parsimony informative sites supporting an alternative topology. (D) Ratio of divergences HC/HG and HC/HO estimated in 100 kb windows.

Assuming that the ancestral effective population size of the X chromosome, *θ*_*X*_, is three quarters that of the ancestral effective population size of the autosomes, the expected amount of ILS on the X chromosome should be 24.08%. The distribution of ILS proportions on the X chromosome is bimodal (Fig 1B) and in stark contrast to the distribution on the autosomes (see also S1 Fig for a breakdown on individual autosomes). One mode represents 63% of the alignment, with a mean proportion of ILS of 21%, close to the expectation of 24% (the 99% confidence interval of the high ILS mode is [17.6%, 24.5%], estimated using parametric bootstrap). The second mode is estimated to represent 37% of the alignment and shows a mean proportion of ILS below 5%. The regions exhibiting low ILS form 8 major segments spread across the X chromosome (Table 1 and Fig 2A) and cover 29 Mb out of a total alignment length of 84 Mb. Region X5 is split in two by the centromeric region, where alignment data are missing. Regions with comparatively low amount of ILS have a higher frequency of genealogy where the human and chimpanzee coalesce within the HC ancestor, while in ILS genealogies, the human and chimpanzee lineages coalesce further back in time, within the HCG ancestor. As a result, low-ILS regions display a lower divergence compared to the rest of the genome. These results are two-fold: (i) they demonstrate that one third of the X chromosome explains the previously reported low divergence of the chromosome, as the remaining two thirds display a divergence compatible with the expectation under a simple model of divergence with an ancestral effective population size equal to three quarters that of the autosomes and (ii) that unique evolutionary forces have shaped the ancestral diversity in the low-ILS regions.

**Table 1:** Low-ILS regions on the X chromosome. Coordinates are given according to the Human genome hg19.

Region	Begin	End	Average ILS
X1	10,241,177	12,619,185	0.035
X2	16,946,047	18,747,389	0.054
X3	19,303,480	22,198,160	0.047
X4	38,344,992	41,272,675	0.062
X5	45,930,478	77,954,462	0.050
X6	99,459,295	111,145,964	0.031
X7	128,232,540	136,796,526	0.034
X8	151,519,514	155,156,362	0.050

### Robustness of ILS estimation

In Scally et al. [21], we independently estimated parameters in non-overlapping windows of 1 Mb, allowing for parameters to vary across the genome. To test whether inference of very low proportions of ILS could result from incorrect parameter estimation, we compared the inferred amount of ILS under alternative parameterizations with that inferred using fixed parameters (either fixing all parameters or fixing speciation time parameters only) along the genome. These alternative parameterizations result in very similar estimates of ILS (S2 Fig and corresponding UCSC genome browser tracks at http://bioweb.me/HCGILSsupp/UCSCTracks/).

We addressed the possibility that our observation is due to a lower power to detect ILS in the identified regions resulting from reduced mutation rate. We counted the number of informative sites supporting each of the three alternative topologies connecting humans, chimpanzees and gorillas in non-overlapping 100 kb windows along the alignment. If the reduction of ILS is due to a lower mutation rate in these regions, we expect to observe a reduction of the amount of parsimony-informative sites supporting all three topologies. While the total frequency of parsimony-informative sites is significantly lower in the low-ILS regions compared with the rest of the genome (0.00270 vs. 0.00276, Fisher's exact test p-value = 1.34e-05), there is a highly significant excess of sites supporting the species topology (0.00229 vs. 0.00210, Fisher's exact test p-value < 2.2e-16) and deficit of sites in these regions supporting ILS topologies (0.00042 vs. 0.00066, Fisher's exact test p-value < 2.2e-16, Fig 2B and 2C), which suggests that the observed reduction of ILS is not the result of a lower mutation rate.

We computed the ratio of human-chimpanzee divergence to human-gorilla divergence and human-orangutan divergence in 100 kb windows. Assuming a constant mutation rate across the phylogeny and constant ancestral effective population sizes along the genome, these ratios should be on average identical between regions from the genome. In regions with reduced ILS, however, this ratio is expected to be lower because of a more recent human-chimpanzee divergence. In agreement with this latter hypothesis, we observe a significant lower ratio of divergences in low-ILS regions (Fig 2D). A lower mutation rate in these regions would explain this pattern only if the reduction is restricted to the human-chimpanzee lineage.

### The effect of background selection on ILS

**Fig 3:**
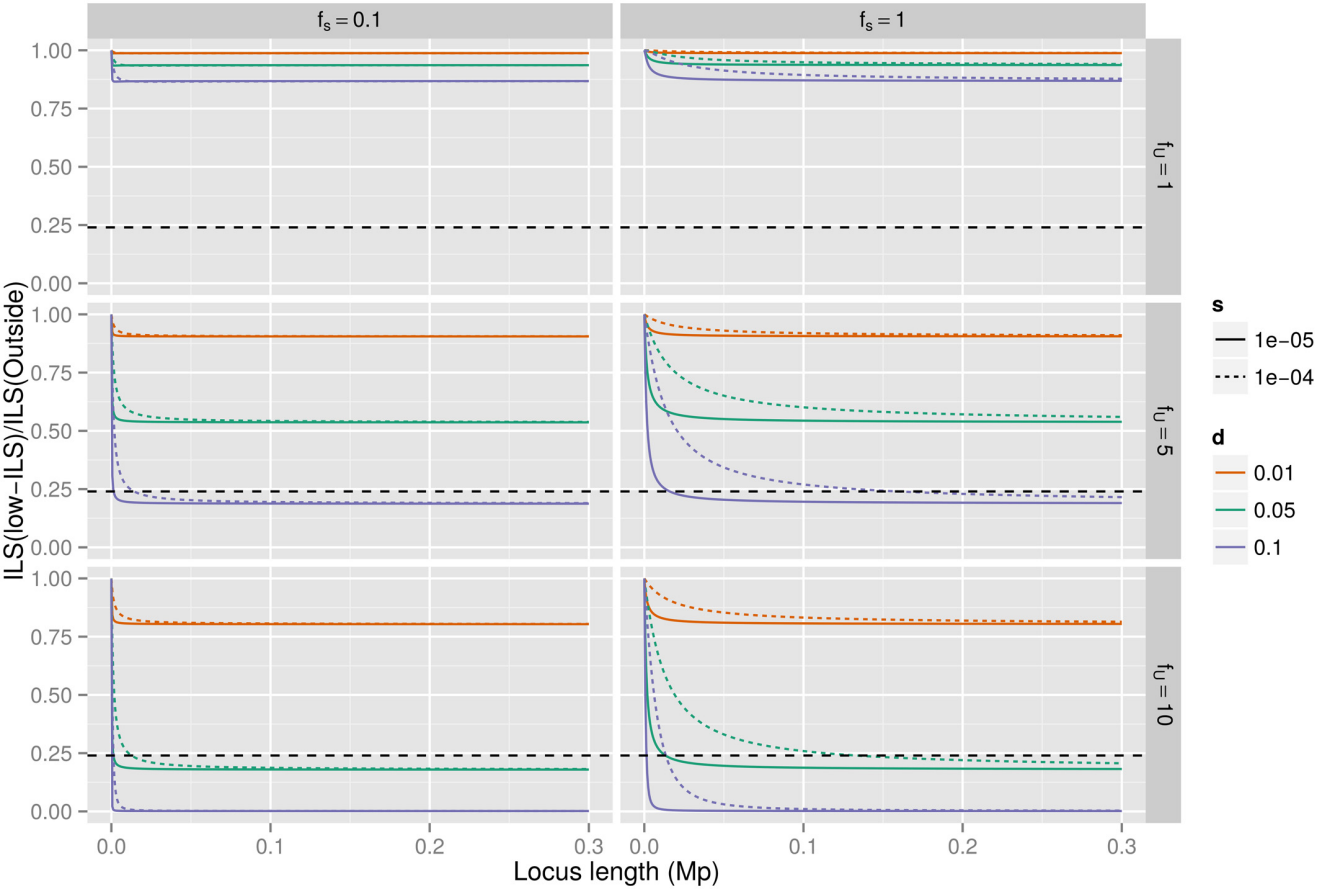
Background selection and ILS. The plots show the ratio of ILS inside the low ILS regions compared to that outside the regions, assuming speciation times of 5.95 mya and 3.7 mya, 20 year generations and that the neutral X effective population size is three quarters that of the autosomes. The colors correspond to different choices of which fraction of mutations are deleterious, varying from 1% to 10%. The different columns correspond to different choices of selection within the low ILS regions—set to either the same as outside or one tenth of the selection strength outside—and different rows show how much more of the regions is under selection compared to outside, either the same or a factor of five or ten. Selection strength is set to either 1e-4 (dotted curve) or 1e-5 (solid curve). The horizontal dashed line represents the observed reduction in ILS of 24% (from 21% ILS outside low-ILS regions to the <5% ILS of low-ILS regions).

Deleterious mutations are continuously pruned from the population through purifying selection, reducing the diversity of linked sequences. Such background selection potentially plays an important role in shaping genetic diversity across the genome [28]. The strength of background selection increases with the mutation rate, with density of functional sites, with decreasing selection coefficient against deleterious mutations, and with decreasing recombination rate [29]. Low-ILS regions display both a 0.6-fold lower recombination rate compared to the rest of the chromosome (1.01 cM/Mb versus 1.62 cM/Mb, Wilcoxon test p-value = 2.2e-07) as well as a two-fold higher gene density—a proxy for the proportion of functional sites (3.1% exonic sites versus 1.5% on average, Wilcoxon test p-value < 2.2e-16). Background selection is therefore both expected to be more common (by a factor of ~2.1 due to more functional sites) and to affect larger regions (by a factor of ~1.8 due to less recombination) in the low-ILS regions. To estimate extent to which this may explain our observations, we used standard analytical results that estimate the combined effect of multiple sites under purifying selection (see Material and Methods). Even if we assume that the proportions of functional sites in the candidate regions is two times higher than the observed number of exon base pairs, and that all mutations at these sites are deleterious with a selection coefficient that maximizes the effect of background selection, the expected proportion of ILS should only be reduced by approximately 10% relative to the level found on the remaining X chromosome (19% ILS compared to 21% ILS). To explain the observed reductions in ILS by background selection alone, unrealistic differences of functional site densities are required (*e*.*g*. 50% inside identified regions and 10% outside, see Figs 3 and S2). As a further line of evidence, we computed the maximal expected reduction of ILS based on the observed density of exonic sites and average recombination rate (see Methods). We find that only 79 of 252 analyzable windows (31%) could be explained by the action of background selection only, an observation incompatible with the hypothesis that background selection is the sole responsible for the widespread reduction of ILS along the X chromosome.

Finally, recombination rate is lower in males than in females. As X chromosomes spend 2/3 of their time in highly recombining females while autosomes spend only half, background selection is expected to be weaker on the X chromosome than on the autosomes. Consequently, in Drosophila where males do not recombine, X chromosomes display a higher than expected diversity [30]. The fact that we do not observe large regions devoid of ILS on the autosomes further argues against background selection as the major force creating the observed large regions with reduced ILS on the X chromosome.

### Selective sweeps and ILS

**Fig 4:**
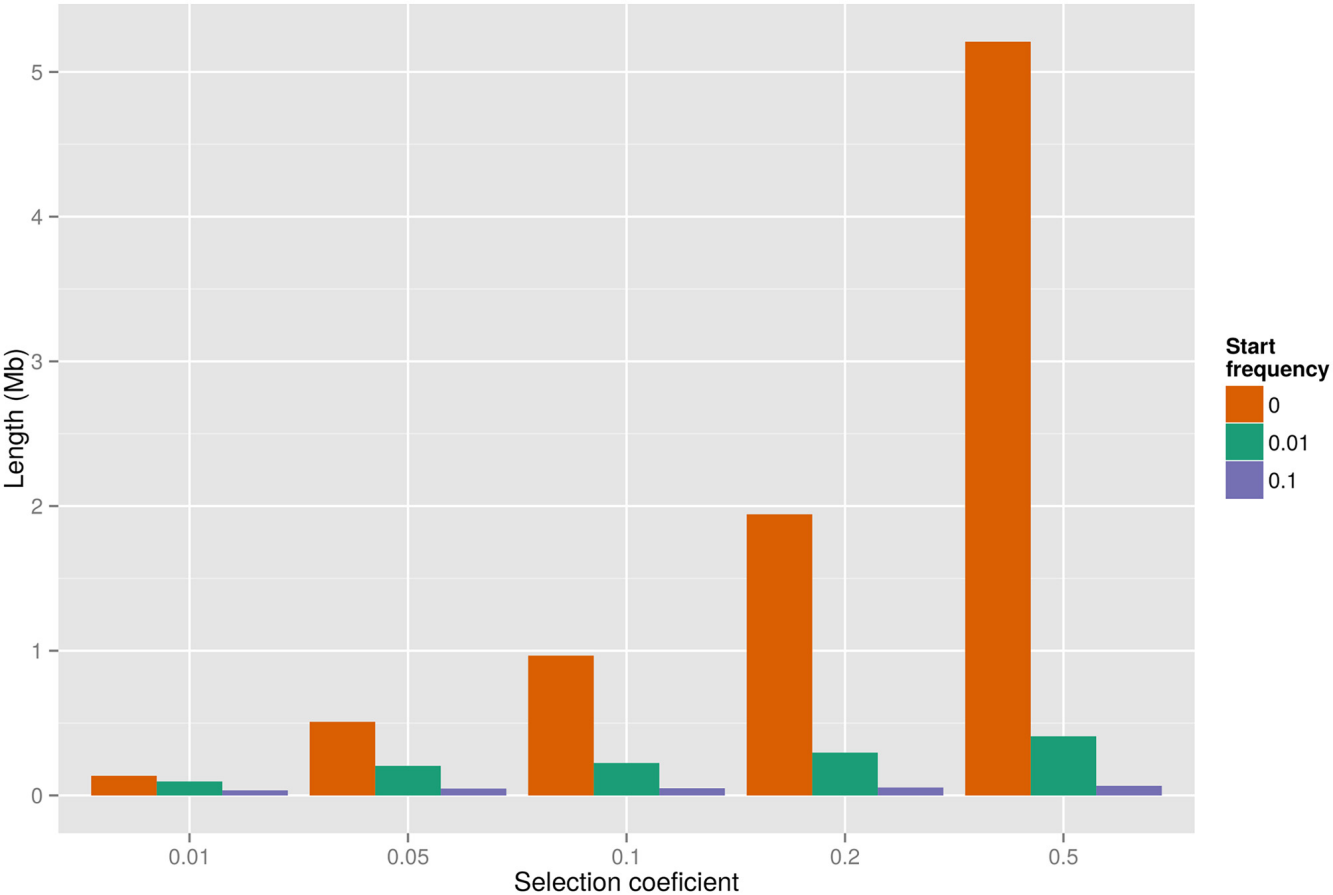
Expected genetic length of the region with less than 5% ILS surrounding a selected mutant with given selection coefficient and start frequency. Lengths are provided assuming a recombination rate of 1 cM/Mb.

Adaptive evolution may also remove linked variation during the process of fixing beneficial variants. In the human-chimpanzee ancestor, such selective sweeps will have abolished ILS at the locus under selection and reduced the proportion of ILS in a larger flanking region. Several sweeps in the same region can thus result in a strong reduction of ILS on a mega-base scale. We simulated selective sweeps in the human-chimpanzee ancestor using a rejection sampling method (see Material and Methods). A single sweep is only expected to reduce ILS to less than 5% on a mega-base wide region if selection coefficients are unrealistically high (s > 0.2), suggesting that several sweeps have contributed to the large-scale depletions of ILS (Figs 4 and S4).

**Fig 5:**
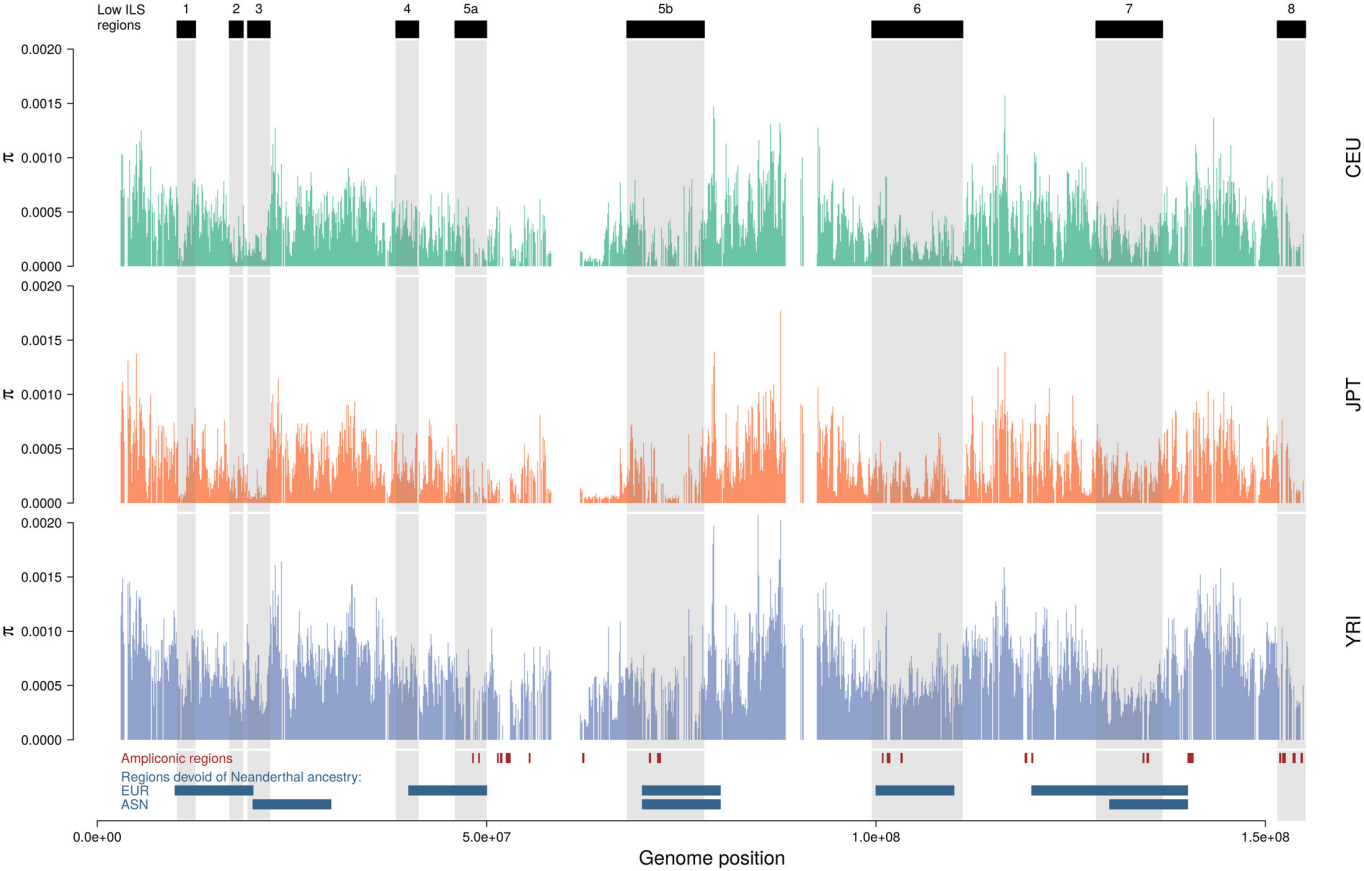
Distribution of nucleotide diversity along the X chromosome of human populations. Nucleotide diversity is computed in 100 kb non-overlapping windows. Ampliconic regions [34] as well as regions with no Neanderthal introgression [14] are shown at the bottom. S5 Fig shows all 14 populations.

If the low-ILS regions are indeed subject to recurrent sweeps, they are expected to also show reduced diversity in human populations. We therefore investigated the patterns of nucleotide diversity in the data of the 1000 Genomes Project [31]. We computed the nucleotide diversity in 100 kb non-overlapping windows along the X chromosome and compared windows within and outside low-ILS regions. Fig 5 summarizes the results for the CEU, JPT and YRI populations (results for all populations are shown in S5 Fig). We find that diversity is significantly reduced in all low-ILS regions compared with the chromosome average (Table 2), and this reduction is on average significantly greater in the Asian and European populations than in the African population (analysis of variance, see Material and Methods). This global difference in magnitude could be explained by phenomena such as sex-biased demography or generation time and population structure during the migration out of Africa [32]. We also compared the eight low-ILS regions separately, and reported differences between regions (Table 3). Plotting population specific diversity across the X chromosome revealed several cases of large-scale depletions of diversity in both Europeans and East Asians. While these depletions affect similar regions, their width differs between populations. This finding suggests that strong sweeps in these regions occurred independently in the European and East Asian population after their divergence less than 100,000 years ago.

**Table 2:** Nucleotide diversity (measured in 100 kb non-overlapping windows) in low-ILS regions in Human populations relative to the X chromosome average outside the low-ILS regions.

Population	Region 1	Region 2	Region 3	Region 4	Region 5	Region 6	Region 7	Region 8
GBR	73% (*)	36% (***)	42% (***)	79% (*)	48% (***)	50% (***)	53% (***)	65% (**)
FIN	78% (.)	35% (***)	45% (***)	81% (.)	48% (***)	47% (***)	54% (***)	59% (***)
CHS	72% (*)	49% (***)	32% (***)	77% (.)	47% (***)	50% (***)	67% (***)	72% (*)
PUR	78% (*)	40% (***)	56% (***)	81% (*)	58% (***)	51% (***)	54% (***)	68% (***)
CLM	75% (*)	43% (***)	47% (***)	76% (*)	55% (***)	54% (***)	58% (***)	70% (**)
IBS	71% (*)	41% (***)	39% (***)	84% (NS)	48% (***)	53% (***)	52% (***)	55% (***)
CEU	73% (*)	36% (***)	39% (***)	78% (*)	51% (***)	47% (***)	54% (***)	62% (***)
YRI	79% (*)	52% (***)	64% (***)	78% (**)	60% (***)	66% (***)	56% (***)	70% (***)
CHB	73% (*)	45% (***)	29% (***)	75% (*)	46% (***)	50% (***)	66% (***)	70% (*)
JPT	76% (.)	47% (***)	32% (***)	81% (NS)	46% (***)	46% (***)	66% (***)	67% (*)
LWK	79% (*)	52% (***)	65% (***)	80% (**)	63% (***)	65% (***)	57% (***)	67% (***)
ASW	77% (*)	50% (***)	65% (***)	77% (**)	65% (***)	65% (***)	54% (***)	69% (***)
MXL	79% (.)	43% (***)	39% (***)	83% (.)	58% (***)	53% (***)	54% (***)	68% (**)
TSI	80% (.)	35% (***)	42% (***)	76% (*)	50% (***)	51% (***)	55% (***)	60% (***)

Stars denote significance of p-values of Wilcoxon tests corrected for multiple testing: 10% (.), 5% (*), 1%(**) < 1% (***).

**Table 3:** Average nucleotide diversity for each population group and low-ILS region, relative to the X chromosome average outside the low-ILS regions. For each region, populations with the same letter code are not significantly different according to Tukey's posthoc test (5% level).

Population	Total	Region 1	Region 2	Region 3	Region 4	Region 5	Region 6	Region 7	Region 8
Africa	64% (a)	78% (a)	51% (a)	64% (a)	78% (a)	63% (a)	65% (a)	55% (a)	70% (a)
America	57% (b)	77% (a)	42% (ab)	47% (b)	80% (a)	57% (b)	53% (b)	55% (a)	70% (a)
Asia	53% (c)	74% (a)	47% (a)	31% (c)	78% (a)	46% (c)	49% (c)	67% (b)	70% (ab)
Europe	53% (c)	75% (a)	37% (b)	41% (b)	80% (a)	49% (d)	50% (c)	54% (a)	60% (b)

## Discussion

Using a complete genome alignment of human, chimpanzee, gorilla and orangutan, we report that the human-chimpanzee divergence along the X chromosome is a mosaic of two types of regions: two thirds of the X chromosome display a divergence compatible with the expectation of an ancestral effective population size of the X equal to three quarters that of the autosome, while one third of the X chromosome shows an extremely reduced divergence, and is virtually devoid of incomplete lineage sorting. We have demonstrated that such diversity deserts cannot be accounted for by background selection alone, but must result from recurrent selective sweeps. We recently reported dramatic reductions in X chromosome diversity in other great ape species that almost exclusively affect areas of the low-ILS regions [18] (see S6 Fig).

If the low-ILS regions evolve rapidly through selective sweeps, they could be among the first to accumulate hybrid incompatibility between diverging populations. Recently, the X chromosome was reported to exhibit many more regions devoid of Neanderthal introgression into modern humans than the autosomes. This suggests an association of negative selection driven by hybrid incompatibility with these X-linked regions [14]. We find a striking correspondence between regions of low ILS and the regions devoid of Neanderthal introgression for European populations (p-value = 0.00021, permutation test) and a marginally significant association with the more introgressed Asian populations (p-value = 0.06721, Fig 5). Taken together, these findings show that the regions on the X chromosome that contributed to hybrid incompatibility in the secondary contact between humans and Neanderthals have been affected by recurrent, strong selective sweeps in humans and other great apes.

The occurrence of a secondary contact between initially diverged populations, one of which diverged into modern chimpanzees and the other admixed with the second to form the ancestral human lineage—the complex speciation scenario of Patterson et al. [23]–is also compatible with our observations: if these regions evolved to be incompatible, the lineages within the regions only came from the ancestral population related to chimpanzees while lineages outside the regions come from both ancestral populations, so that we would also expect to see reduced ILS within the regions and not outside the regions. However, such a complex speciation scenario does not explain the observed large-scale reductions of diversity in extant species. Conversely, a scenario consisting only of recurrent sweeps would explain both the divergence patterns along the human and chimpanzee X chromosomes and the reduction of extant diversity, without the need for secondary introgression.

To explain the occurrence of recurrent selective sweeps in the lineage of great apes, we propose a hypothesis that may account for the generality of our findings: Deserts of diversity may arise via meiotic drive, through which fixation of variants that cause preferential transmission of either the X or Y chromosome produces temporary sex ratio distortions [17]. When such distortions are established, mutations conferring a more even sex ratio will be under positive selection. Potential candidates involved in such meiotic drive are ampliconic regions, which contain multiple copies of genes that are specifically expressed in the testis. These genes are postmeiotically expressed in mice, and a recent report suggests that the Y chromosome harbors similar regions [33]. Fourteen of the regions identified in humans [34] are included in our alignment, 11 of which are located in low-ILS regions (Figs 2 and 5), representing a significant enrichment (p-value = 0.01427, permutation test), a result which is even more significant when regions in the centromeric region are included (p-value = 0.00642).

Whatever the underlying mechanism, our observations demonstrate that the evolution of X chromosomes in the human chimpanzee ancestor, and in great apes in general [18], is driven by strong selective forces. The striking overlap between the low-ILS regions we have identified and the Neanderthal introgression deserts identified by Sankararaman et al. [14] further hints that these forces could be driving speciation.

## Materials and Methods

### Genome alignment and data pre-processing

The Enredo/Pecan/Ortheus genome alignment of the five species human, chimpanzee, gorilla, orangutan and macaque from Scally et al. [21] was used as input. In order to remove badly sequenced and / or ambiguously alignment regions, we filtered the input 5-species alignments using the MafFilter program [35]. We sequentially applied several filters to remove regions with low sequence quality score and high density of gaps. Details on the filters used can be found in the supplementary material of Scally et al. [21]

### Inference of incomplete lineage sorting

The divergence of two genomes depends on both the mutation rate and underlying demographic scenario. With a constant mutation rate u and simple demography (constant sized panmictic population evolving neutrally), the time to the most recent common ancestor of two sequences sampled from different species is given by a constant species divergence, *τ* = *T*.*u*, and an ancestral coalescence time following an exponential distribution with mean *θ* = 2.*Ne*_*A*_.*u*, where T is the number of generations since species divergence and Ne_A_ is the ancestral effective population size [22,36]. For species undergoing recombination, a single individual genome is a mosaic of segments with distinct histories, and therefore displays a range of divergence times [22,23,37]. When two speciation events separating three species follow shortly after each other, this variation of genealogy can lead to incomplete lineage sorting (ILS), where the topology of gene trees do not correspond to that of the species tree [22,26]. Reconstructing the distribution of divergence along the genome and the patterns of ILS allows inference of speciation times and ancestral population sizes. We used the CoalHMM framework to infer patterns of ILS along the X chromosome. Model fitting was performed as described in [21]. ILS was estimated using posterior decoding of the hidden Markov model as the proportions of sites in the alignment which supported one of the (HG),C or (CG),H topologies. All parameter estimates can be visualized in the UCSC genome browser using tracks available at http://bioweb.me/HCGILSsupp/.

### Distribution of ILS

For the autosomal distribution of ILS, we fitted a skewed normal distribution (R package 'sn' [38]) using the *fitdistr* function from the MASS package for R. For the X chromosome ILS distribution, we fitted a mixture of gamma and Gaussian distributions. The mixed distribution follows a normal density with probability p, and a gamma density with probability 1-p. In addition to p, the mixed distribution has four parameters: the mean and standard deviation of the Gaussian component, and the shape and rate of the gamma component. The L-BFGS-B optimization method was used to account for parameter constraints. Resulting parameter estimates are 0.209 for the mean of the Gaussian component, 0.066 for the standard deviation of the Gaussian component, 4.139 for the alpha parameter (shape) of the gamma component, 83.369 for the beta parameter (rate) of the gamma component, and p = 0.632. The mean of the gamma component is alpha / beta = 0.0497, that is, less than 5% ILS. We compared the resulting fit with a mixture of skewed normal distributions, which has two extra parameters compared to a Gamma-Gaussian mixture, and found that the skew of the higher mode is very close to zero, while the Gamma distribution offered a better fit of the lower mode. We used a parametric bootstrap approach to estimate the confidence interval of the proportion of ILS for the mean of the normal component of the mixed distribution. We generated a thousand pseudo-replicates by sampling from the estimated distribution, and we re-estimated all parameters from each replicate in order to obtain their distribution. Replicates where optimization failed were discarded (40 out of 1000).

### Characterization of low-ILS regions

In order to characterize the patterns of ILS at a finer scale, we computed ILS in 100 kb windows sliding by 20 kb along the posterior decoding of the alignment. To exhibit regions devoid of ILS, we selected contiguous windows with no more than 10% of ILS each. Eight of these regions were greater than 1 Mb in size, and their resulting amount of ILS is less than 5% on average (Table 1). The coordinates of these regions were then translated according to the human hg19 genome sequence. These data are available as a GFF file for visualization in the UCSC genome browser at http://bioweb.me/HCGILSsupp/.

### Reduction in ILS by background selection

Background selection reduces diversity by a process in which deleterious mutations are continuously pruned from the population. The strength of background selection in a genomic region is determined by the rate at which deleterious mutations occur, *U*, the recombination rate of the locus, *R*, and the strength of negative selection on mutants, *s*. We consider the diversity measure,*π*(the pairwise differences between genes) which in a randomly mating population is linearly related to the effective population size. If *π*_*0*_ denotes diversity in the absence of selection and *π* the diversity in a region subject to background selection, then the expected reduction in diversity is given by

$$\frac{\pi }{\pi_{0}}=exp\left(\frac{-U}{s+R}\right)$$ (1)

(see Durrett [39] equation (6.24))

The rates *U* and *R* are both functions of the locus length (*U* = *uL* and *R* = *rL*) where *r* denotes the per-nucleotide-pair recombination rate, *u* the per-nucleotide deleterious rate, and *L* the length of the locus. To investigate if background selection can explain the observed reductions in ILS we must compute the expected reduction in diversity in the low-ILS regions relative to the reduction in the remaining chromosome. A larger reduction in low-ILS regions may be caused by weaker negative selection, higher mutation rate, lower recombination rate, and larger proportion of functional sites at which mutation is deleterious. To model the variation of these parameters inside and outside low-ILS regions we simply add a factor to each relevant variable. The relative reduction can thus be expressed as:

$$\frac{\pi_{low-ILS}}{\pi_{genome}}=\frac{exp\left(\frac{U}{s+R}\right)}{exp\left(\frac{f_{u}.U}{f_{s}.s+f_{R}.R}\right)}$$ (2)

The recombination rate, *R*, and the factor, *f*_*R*_, can be obtained from the deCODE recombination map [40]. We computed the average deCODE recombination rate, as well as the proportion of sites in exons (as a measure of selective constraint) in non-overlapping 100 kb along the human X chromosome.

The recombination rate average outside the low ILS regions is 1.62 cM/Mb and the recombination rate inside the regions is 1.01 cM/Mb which gives us *f*_*R*_ = 0.6. For the remaining parameters, *s* and *U*, we need to identify realistic values outside the low-ILS regions. Background selection is stronger when selection is weak, but the equation is not valid for very small selection values where selection is nearly neutral. Once *s* approaches 1/*N*_*e*_, we do not expect any background selection. Most stimates of effective population sizes, *N*_*e*_, in great apes are on the order 10,000–100,000 and this puts a lower limit on relevant values of *s* at 10^−4^–10^−5^. To conservatively estimate the largest possible effect of background selection we explore this range of selection coefficients: *s* = 10^−4^ and *s* = 10^−5^ and allow the selection inside the low ILS regions to be one tenth (*fs* = 0.1) of that outside. For *U* values outside low-ILS regions we assume the mean human mutation rate, estimated to be *1*.*2*·10^−8^ per generation [41]. To obtain the rate of deleterious mutation we must multiply this with the proportion of sites subject to weak negative selection, *d*. Although this proportion is subject to much controversy it is generally believed to be between 3% and 10% [42]. However, as explained below we explore values up to 100% inside the low-ILS regions.

We assessed the relative diversity for combinations of *s* and *d* values (S3 Fig). Each cell represents a combination of parameter values for *s*, *d*, *f*_*U*_ and *f*_*s*_. The reduction of diversity *Δπ* translates into reduction of ILS, *ΔILS*(Fig 3). Assuming the time between speciation events, the generation time and population size reported in Scally et al. [21] (*ΔT* = 2,250,000 years, g = 20) ILS is given by

$$ILS=\frac{2}{3}exp\left(\frac{-\Delta T/g}{3/4\times \pi }\right)$$ (3)

and the relative ILS is given by

$$\frac{ILS}{ILS_{0}}=exp\left(\frac{\Delta T/g}{3/4}\left(\frac{1}{\pi_{0}}-\frac{1}{\pi }\right)\right).$$ (4)

For the most extreme parameter values, we see a relative reduction in ILS of nearly 100%. In these cases, however, 100% of the nucleotides within low-ILS regions are under selection. In the cases where 25% of the nucleotides in the low-ILS regions are under selection compared to 5% outside (*f*_*U*_ = 5, *d* = 0.05), the regions retain more than half of the diversity seen outside the regions.

We further computed the expected reduction of ILS due to background selection in 100 kb windows located in low-ILS regions using (eq 4). For each window, we computed the frequency of sites in exons and the average deCODE recombination rate. We further assumed a selection coefficient *s* = 10^−5^ and allow the selection inside the low ILS regions to be one tenth (*fs* = 0.1). Out of 285 windows located in low-ILS regions, we could estimate the maximal reduction of ILS due to background selection in 252 windows for which a deCODE recombination estimate was available. In 79 of these windows only the expected reduction matched the observed one of 0.20.

### Simulation of ancient selective sweeps

To assess how hard and soft sweeps in the human-chimpanzee ancestor can have reduced the proportion of ILS we simulated sweeps for different combinations of selection coefficients, s, and frequencies of the selected variant at the onset of selection, *f*. Frequency trajectories of selected variants are obtained using rejection sampling to obtain trajectories that fix in the population. Trajectories used to simulate hard sweeps begin at one and proceed to fixation at 2N * 3/4 by repeated binomial sampling with probability parameter N_mut_/(N_mut_ + (N − N_mut_)(1-s)), where N_mut_ is the number of selected variants in the previous generation. We use a human-chimpanzee speciation time of 3.7 Myr, a human-gorilla speciation time of 5.95 Myr, a human-chimpanzee effective population size of 73,200 as reported in [21], assuming a mutation rate of 1e-9 and a generation time of 20 years. Trajectories used to simulate soft sweeps are constructed by joining two trajectories. If f is the frequency of the variant at the onset of selection F = f * 2N * 3/4 is the number of variants. We first sample a trajectory that represents the time before the onset of selection. This trajectory is required to reach F at least once before it fixes or is lost, and is truncated randomly at one of the points where it passes the value F. The truncated trajectory is then appended with a trajectory under selection that begins at F and proceeds to fixation.

In each simulation we consider a sample of two sequences that represent 10 cM. As the effect of the sweep is symmetric we only simulate one side of the sweep. We then simulate backwards in the Wright-Fisher process with recombination allowing at most one recombination event per generation per lineage but allowing mergers of multiple lineages expected to occur in strong sweeps. The simulation proceeds until all sequence segments have found a most recent common ancestor (TMRCA). For each combination of parameters s and f we perform 1,000 simulations and the mean TMRCA is computed in bins of 10 kb.

In each simulation individual sequence segments are called as ILS with probability 2/3 if the TMRCA exceeds the time between the speciation events. The width of the region showing less than 5% ILS is then computed for each simulation. In Figs 4 and S3 a recombination rate of 1 cM/Mb is assumed to translate to physical length.

### Comparing diversity between human populations

We computed the nucleotide diversity in 100 kb non-overlapping windows along the X chromosome for the 14 populations from the 1,000 genomes project. The windows in each low-ILS region were compared to windows outside the regions using a Wilcoxon test with correction for multiple testing [43] (Table 2). We computed the relative nucleotide diversity in the 1,298 windows located in low-ILS regions by dividing by the average of the rest of the X chromosome. Each population was further categorized according to its origin, Africa, America, Asia or Europe [31]. A linear model was fitted after Box-Cox transformation:

BoxCox[RelativeDiversity] ~ (Region / Window) * (PopulationGroup / Population)

where Window is the position of the window on the X chromosome, and is therefore nested in the (low-ILS) Region factor. Analysis of variance reeals a highly significant effect of the factors Region and Window (p-values < 2e-16), PopulationGroup (p-value < 2e-16) and their interactions (p-value < 2e-16). The nested factor Population however was not significant, showing that the patterns of relative diversity within low-ILS regions are similar between populations within groups. A Tukey's Honest Significance Difference test (as implemented in the R package 'agricolae') was performed on the fitted model and further revealed that European and Asian diversity are not significantly different, while they are different from African and American diversity.

### Association with ampliconic regions and Neanderthal introgression-free regions

In order to test the association of low-ILS regions with other genomic features, we developed a Monte-Carlo simulation procedure. In such a test, we wanted to compare a set of "reference" intervals with a set of "query" intervals. The null hypothesis is that the query intervals are independent of the reference intervals. We use the size of the overlap of the two sets of intervals as a statistic. During the randomization procedure, the set of query intervals is shuffled, so that each interval is conserved in length, only the relative order and positions of intervals are changed. Intervals are not allowed to overlap, so that the size of the query set is constant through simulations and identical to the observed one. The distance between two intervals is however allowed to be zero. For each simulation, the size of the overlap with the reference set of intervals is computed. A p-value is calculated by counting the number of simulations with an overlap at least equal to the observed one. In order to randomize intervals, we developed the following procedure: 1) compute the total size S of the chromosome not included in any interval of the query set; 2) draw n breakpoints uniformly between 0 and S, where n in the number of intervals in the query set; 3) insert randomly one query interval at each breakpoint. This procedure has the advantage that it keeps the structure of the reference set, so that the putative auto-correlation of reference intervals along the genome is accounted for. The 'intervals' R package was used for handling intervals and computing their overlap, and 100,000 randomizations were performed for each test.

We applied the randomization test to the two sets of Neanderthal introgression free regions for European and Asian populations, as well as for the ampliconic regions. The coordinates of ampliconic regions tested in [34] were translated to hg19 using the liftOver utility from UCSC. Fourteen regions were included in our alignment. For all tests, the set of low-ILS regions was used as a query set. For ampliconic regions, we performed a second test where ampliconic regions located close to the centromere and not included in our alignment were discarded.

## Supporting Information

S1 Fig Distribution of ILS for each chromosome. Lines correspond to fitted densities of a normal distribution (blue), a skewed normal distribution (green) and a mixture of gamma + normal distributions (orange). The value of alpha, indicated on each plot corresponds to the value of this parameter from the fit of the skewed normal distribution. Alpha = 0 corresponds to a normal distribution, making the blue and green curves indistinguishable. The p parameter corresponds to the proportion of the gamma component of the mixed distribution. If p is zero, then the mixed distribution could not be fitted (absence of orange curve).
(PDF)

S2 Fig Effect of parameter estimation on ILS inference on the X chromosome alignment. ILS is computed in 1 Mb alignments. The x-axis shows the inferred amount of ILS when model parameters are estimated independently on each alignment (free parameters). The left graph shows the amount of ILS inferred when all model parameters are assumed constant along the X chromosome, estimated from the full chromosome alignment (fixed parameters). The right graph shows the amount of ILS inferred when only the speciation times are considered constant along the chromosome; ancestral population sizes and recombination rate are allowed to vary and are estimated independently for each alignment.
(PDF)

S3 Fig Background selection and diversity. The plots show the ratio of nucleotide diversity inside the low ILS regions compared to that outside the regions, assuming speciation times of 5.95 mya and 3.7 mya, 20 year generations and that the neutral X effective population size is three quarters that of the autosomes. Rest of legend is as in Fig 3.
(PDF)

S4 Fig Distribution of the genetic length of the region with less than 5% ILS extending away from a selected mutant. Each panel shows the distribution for a combination of selection coefficient, and frequency of the mutant at the onset of selection. Each sub-plot is based on 1,000 simulations.
(PDF)

S5 Fig Distribution of nucleotide diversity along the X chromosome for the 14 populations from the 1000 Genomes Project. Nucleotide diversity is computed in 100 kb non-overlapping windows. Ampliconic regions [34] as well as regions with no Neanderthal introgression [14] are shown at the bottom.
(PDF)

S6 Fig Nucleotide diversity of 100 kb windows in low-diversity regions (< 20% of species average) in great apes. Blue bars represent low-ILS regions identified in this study. B: Bonobo, CC: Central chimpanzee, EC: Eastern chimpanzee, WC: Western chimpanzee, NC: Nigerian chimpanzee, WLG: Western lowland gorilla, SO: Sumatran orangutan, BO: Bornean orangutan.
(PDF)
