# Supplementary figures and images for: Strong Selective Sweeps on the X Chromosome in the Human-Chimpanzee Ancestor Explain Its Low Divergence
Source: PLoS Genet. 2015 Aug 14;11(8):e1005451. doi: 10.1371/journal.pgen.1005451 (PMC4537231; doi:10.1371/journal.pgen.1005451)

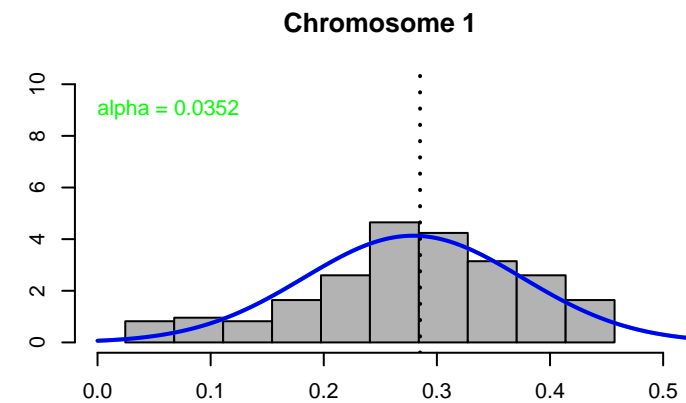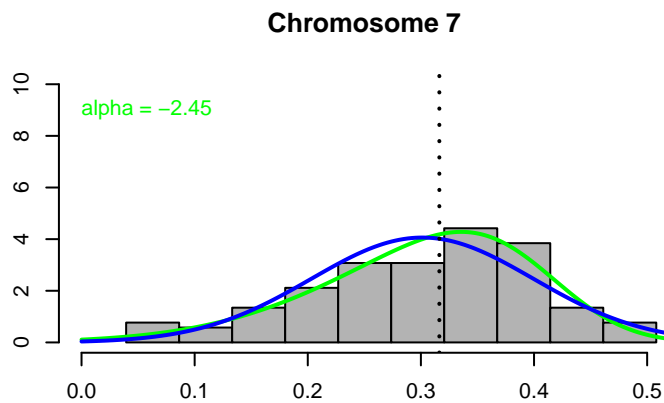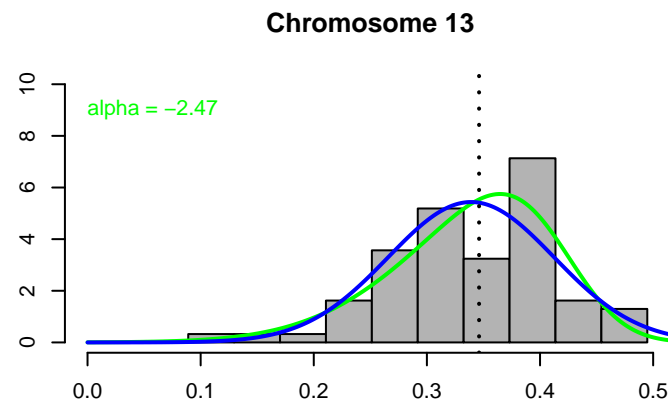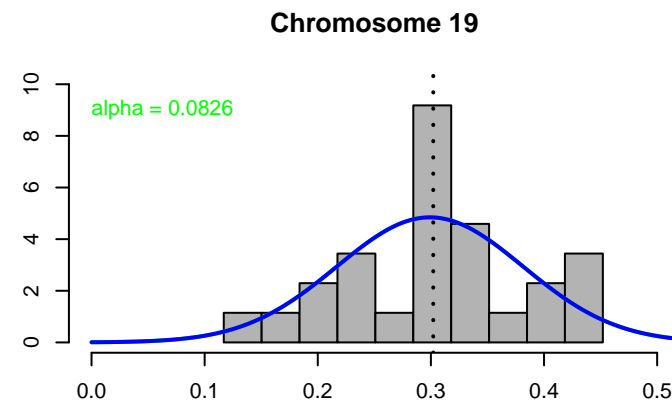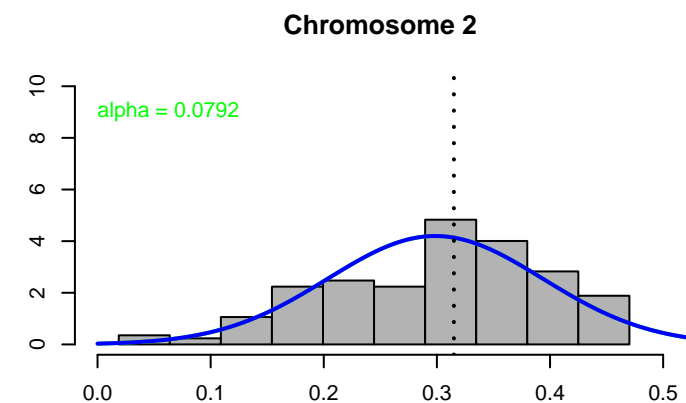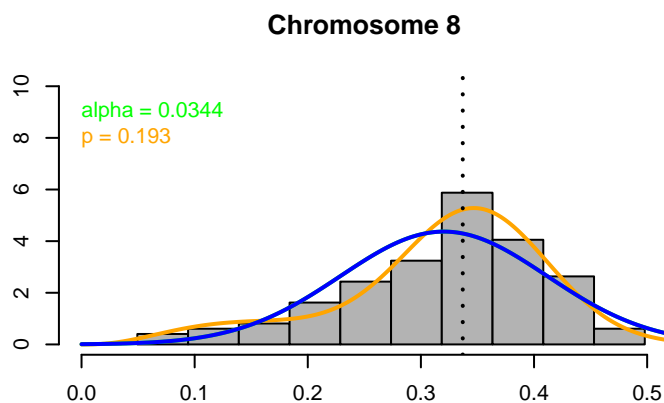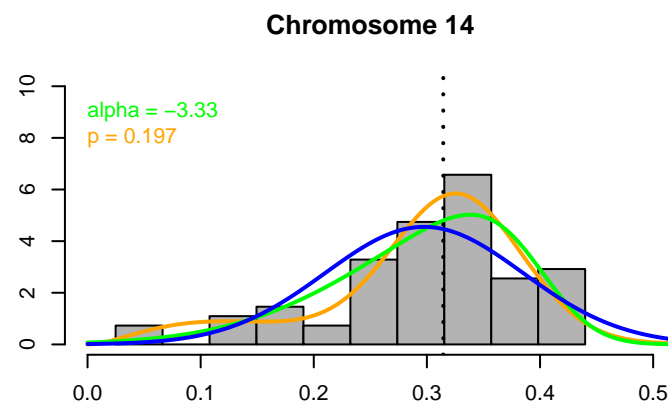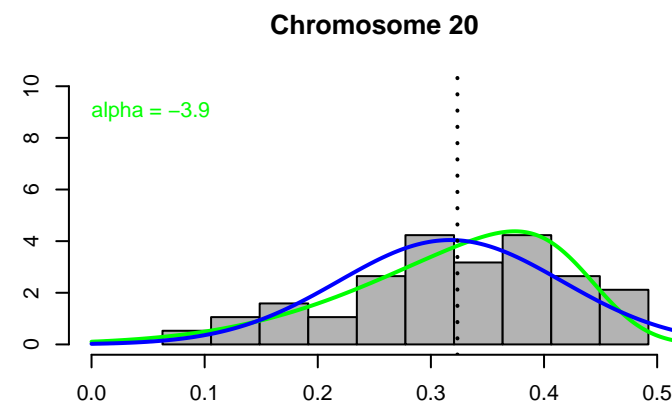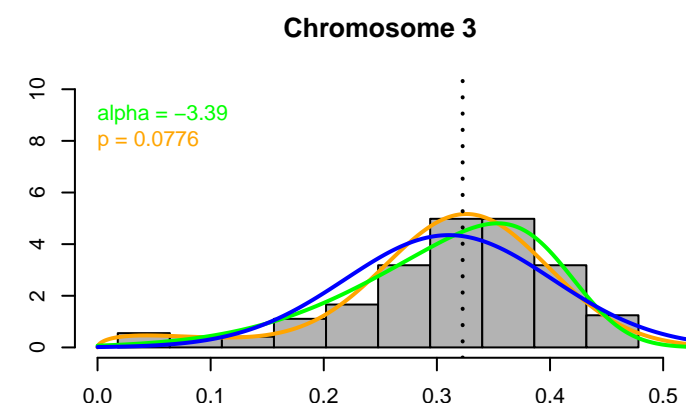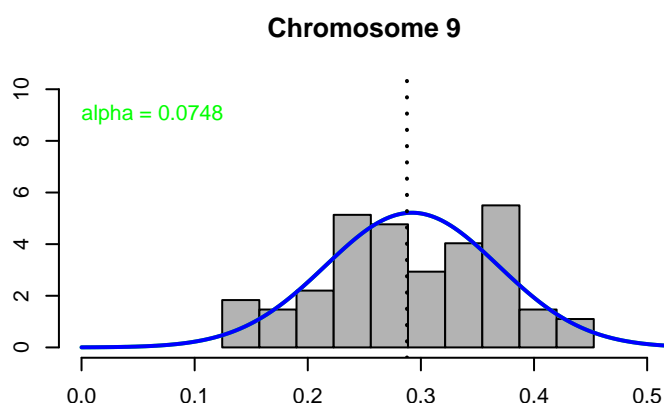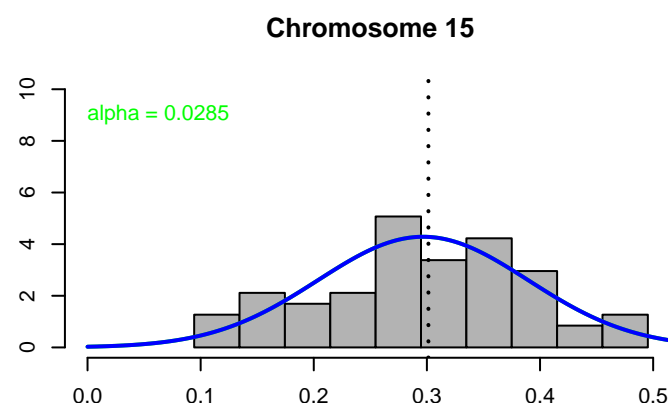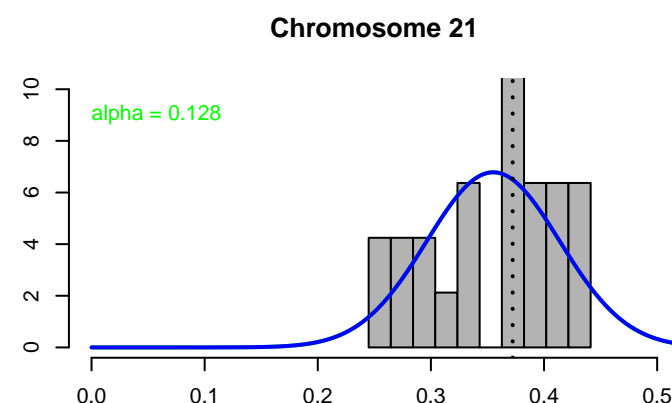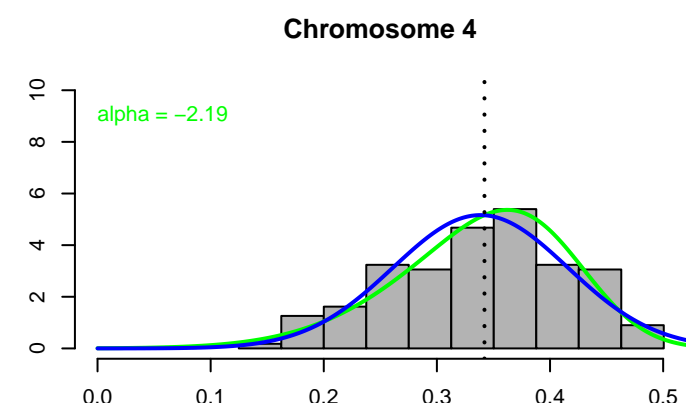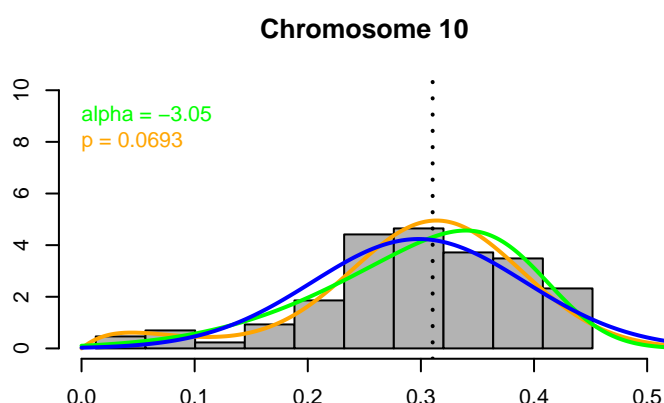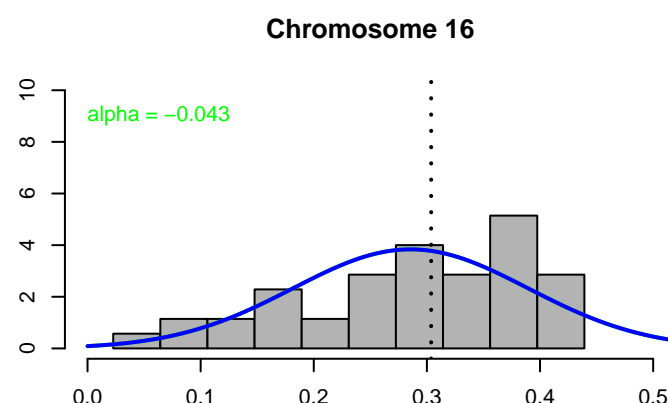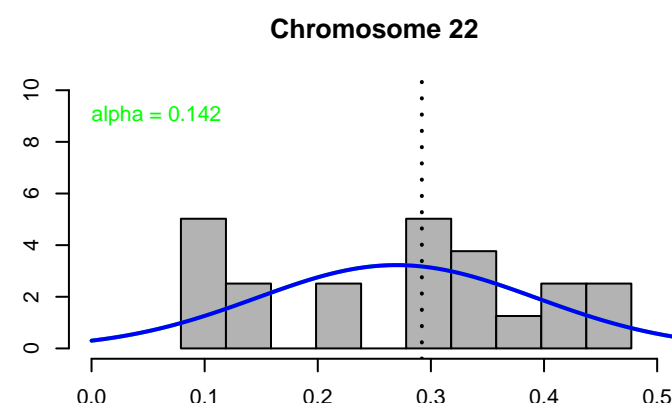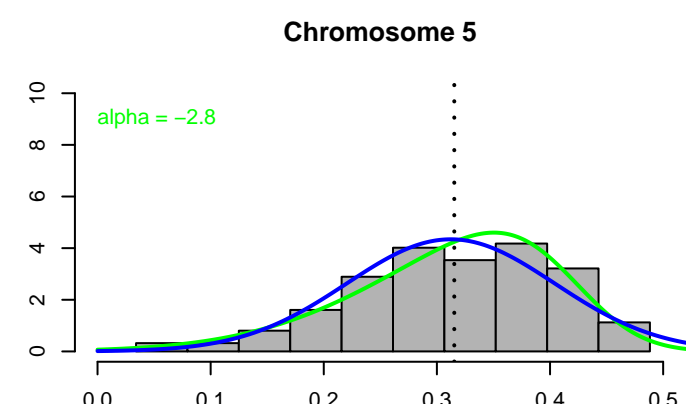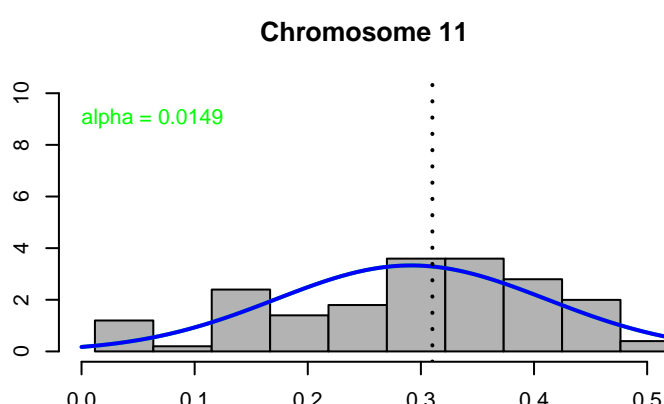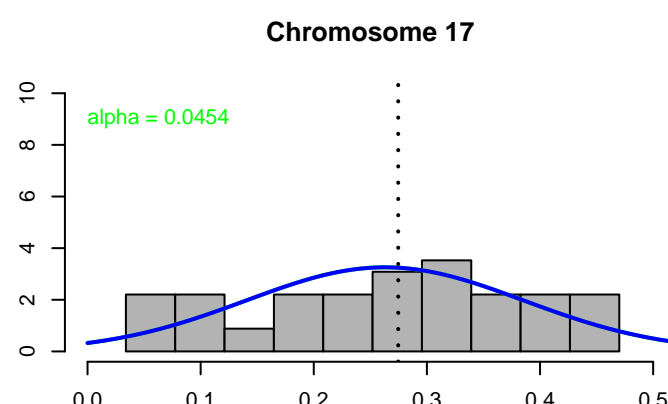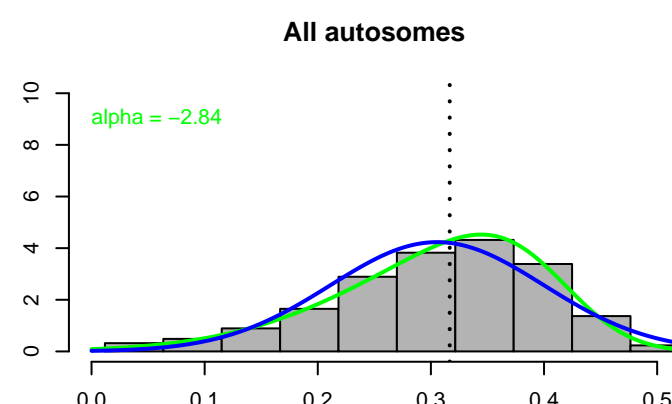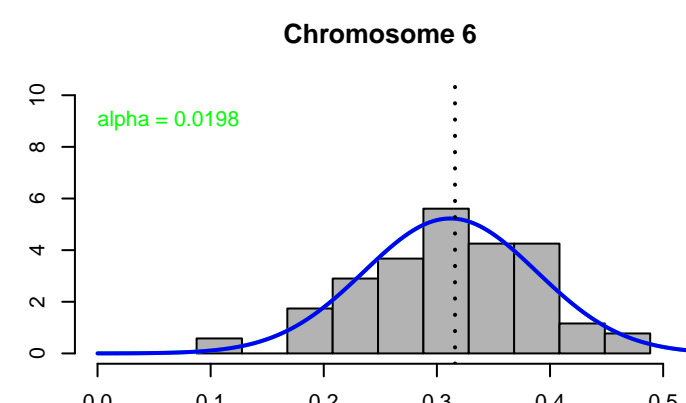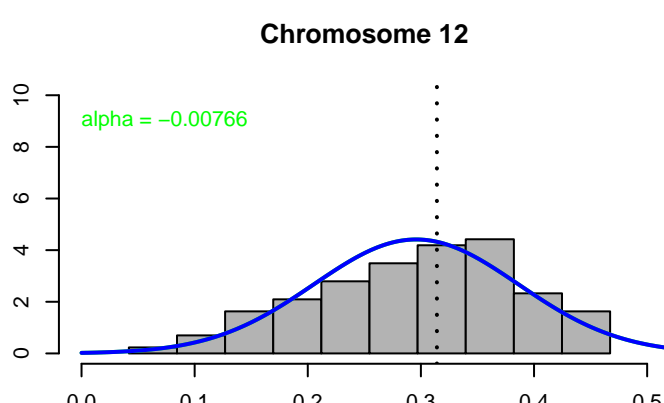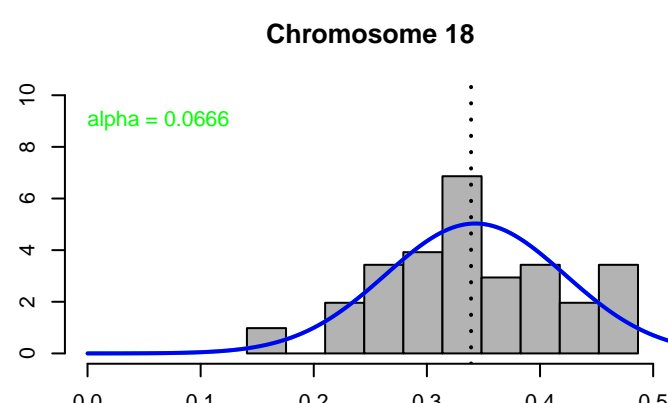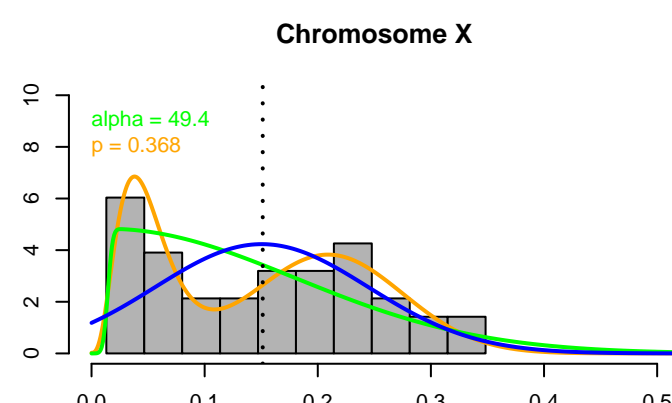

Supplement: S1 Fig — Lines correspond to fitted densities of a normal distribution (blue), a skewed normal distribution (green) and a mixture of gamma + normal distributions (orange). The value of alpha, indicated on each plot corresponds to the value of this parameter from the fit of the skewed normal distribution. Alpha = 0 corresponds to a normal distribution, making the blue and green curves indistinguishable. The p parameter corresponds to the proportion of the gamma component of the mixed distribution. If p is zero, then the mixed distribution could not be fitted (absence of orange curve). (PDF) [file pgen.1005451.s001.pdf]

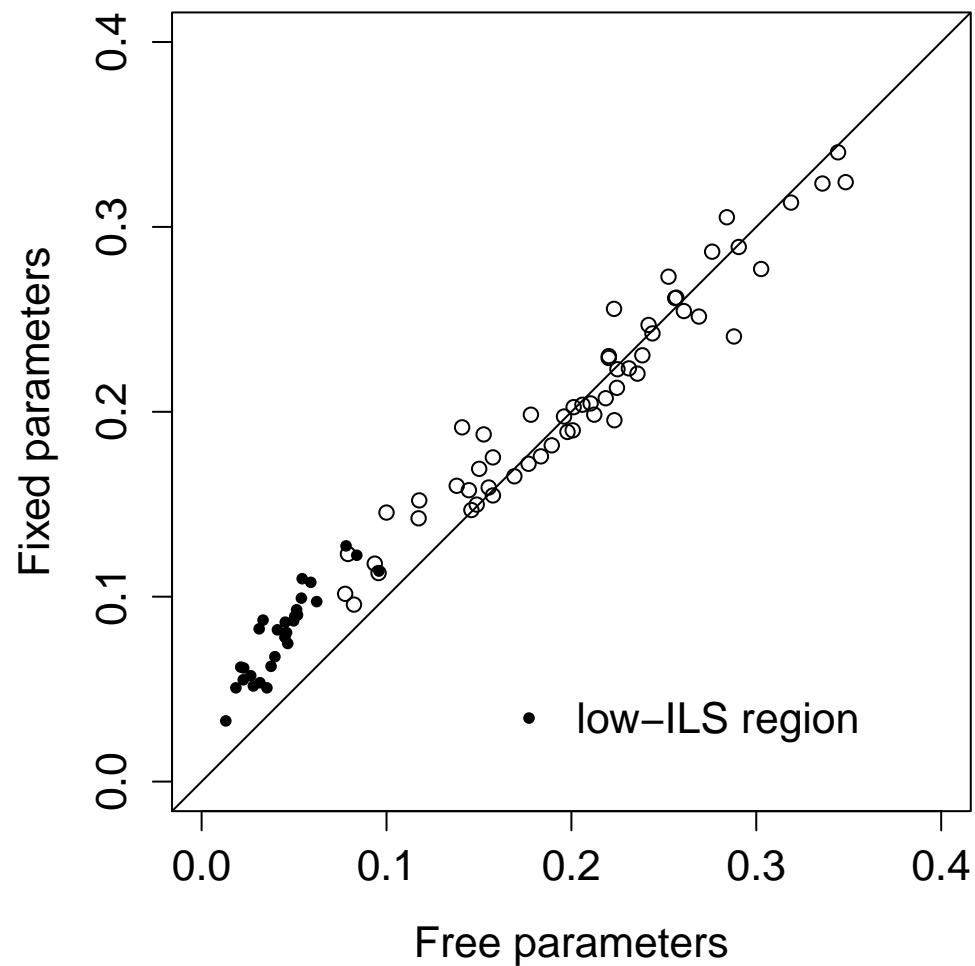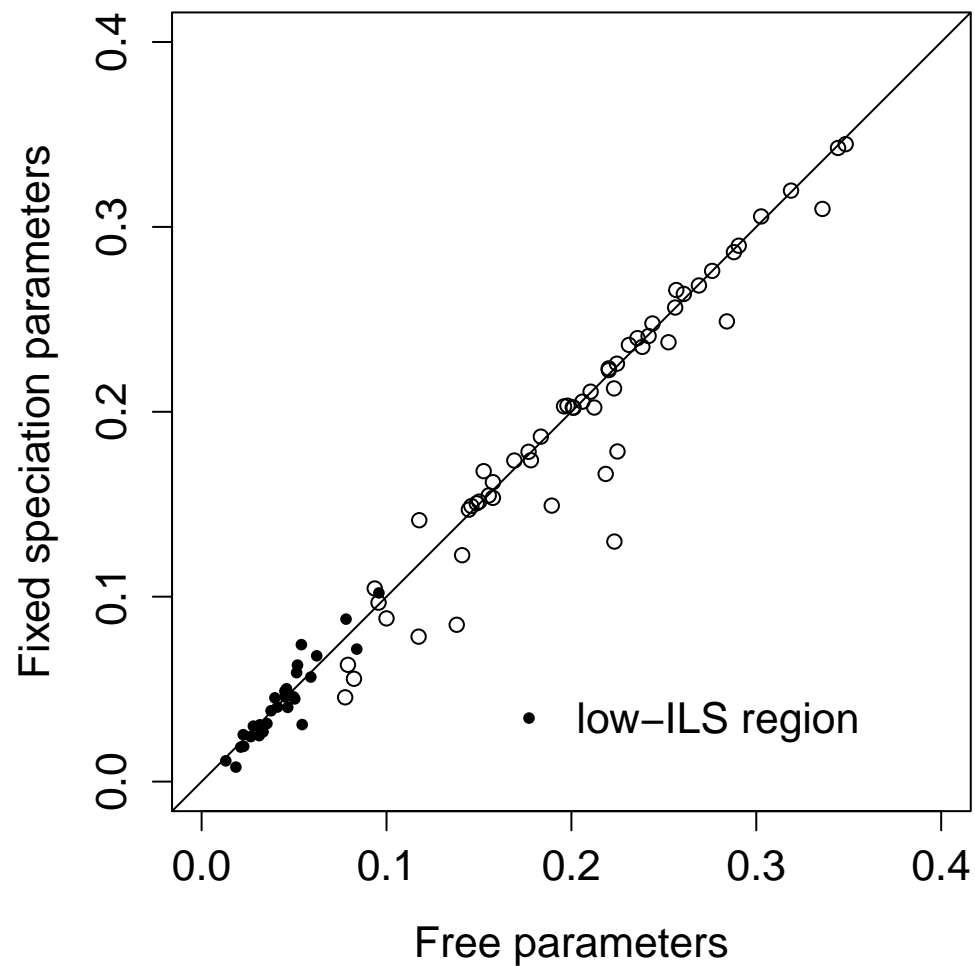

Supplement: S2 Fig — ILS is computed in 1 Mb alignments. The x-axis shows the inferred amount of ILS when model parameters are estimated independently on each alignment (free parameters). The left graph shows the amount of ILS inferred when all model parameters are assumed constant along the X chromosome, estimated from the full chromosome alignment (fixed parameters). The right graph shows the amount of ILS inferred when only the speciation times are considered constant along the chromosome; ancestral population sizes and recombination rate are allowed to vary and are estimated independently for each alignment. (PDF) [file pgen.1005451.s002.pdf]

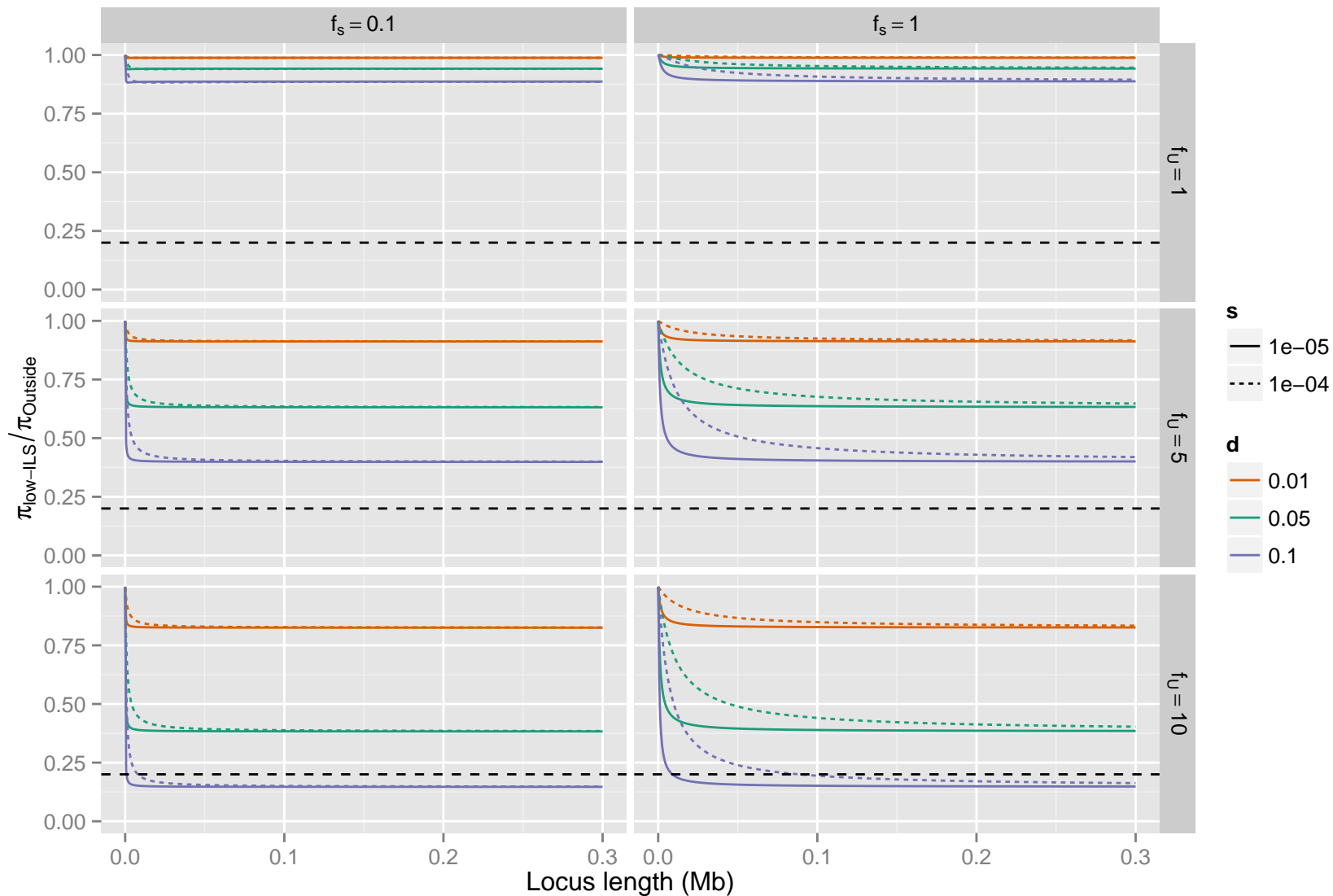

Supplement: S3 Fig — The plots show the ratio of nucleotide diversity inside the low ILS regions compared to that outside the regions, assuming speciation times of 5.95 mya and 3.7 mya, 20 year generations and that the neutral X effective population size is three quarters that of the autosomes. Rest of legend is as in Fig 3. (PDF) [file pgen.1005451.s003.pdf]

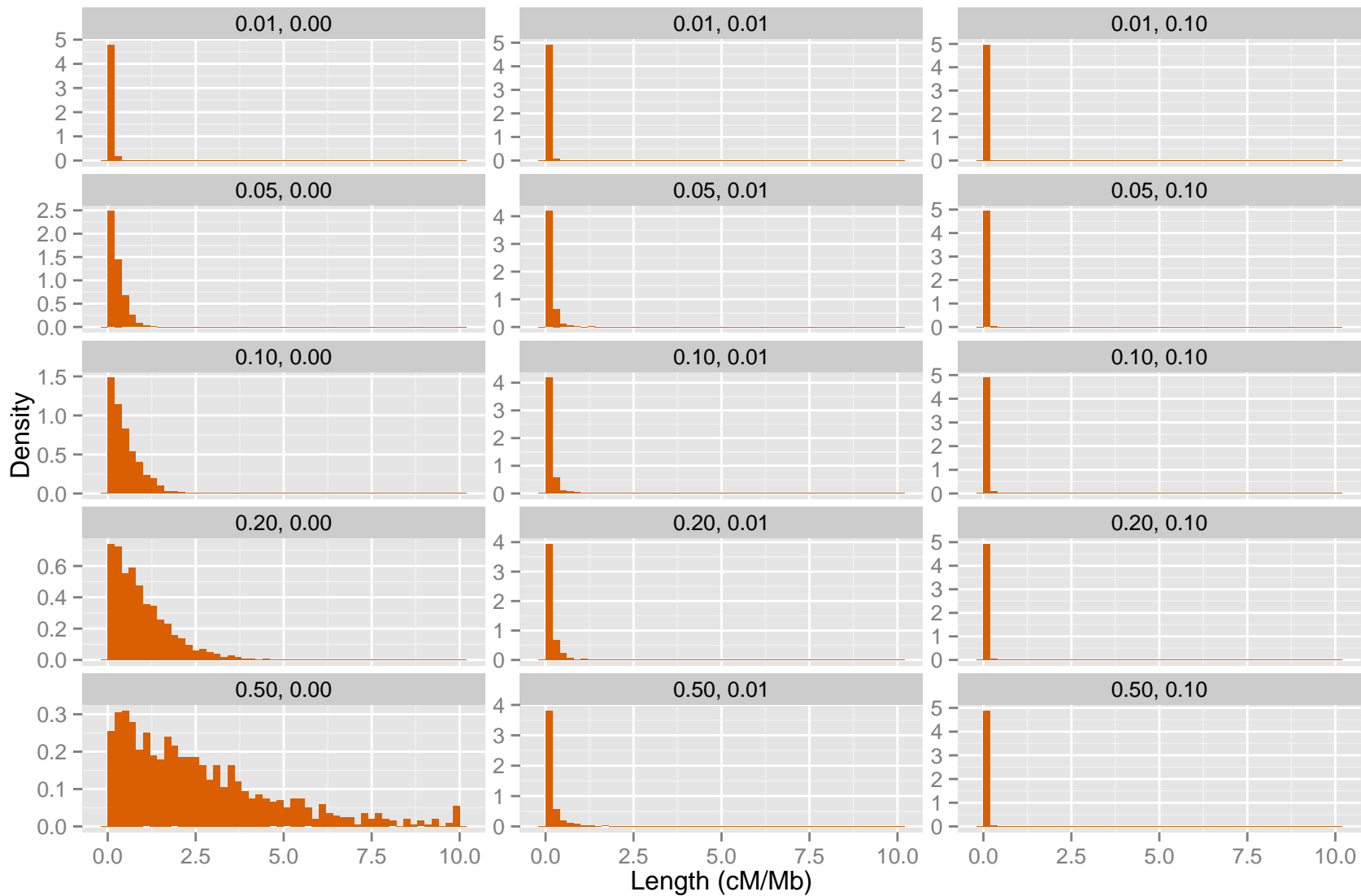

Supplement: S4 Fig — Each panel shows the distribution for a combination of selection coefficient, and frequency of the mutant at the onset of selection. Each sub-plot is based on 1,000 simulations. (PDF) [file pgen.1005451.s004.pdf]

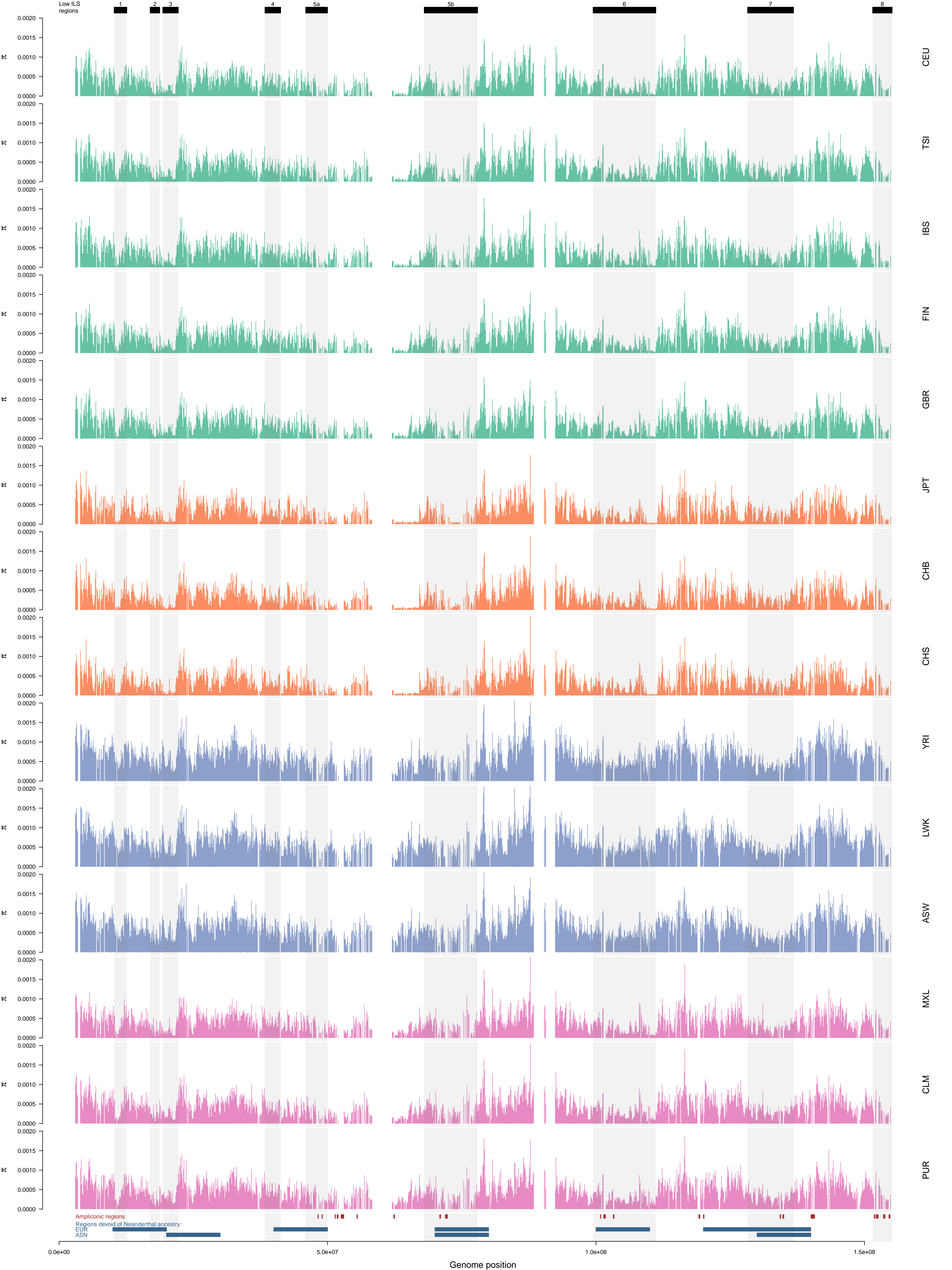

Supplement: S5 Fig — Nucleotide diversity is computed in 100 kb non-overlapping windows. Ampliconic regions [34] as well as regions with no Neanderthal introgression [14] are shown at the bottom. (PDF) [file pgen.1005451.s005.pdf]

Regions with low diversity

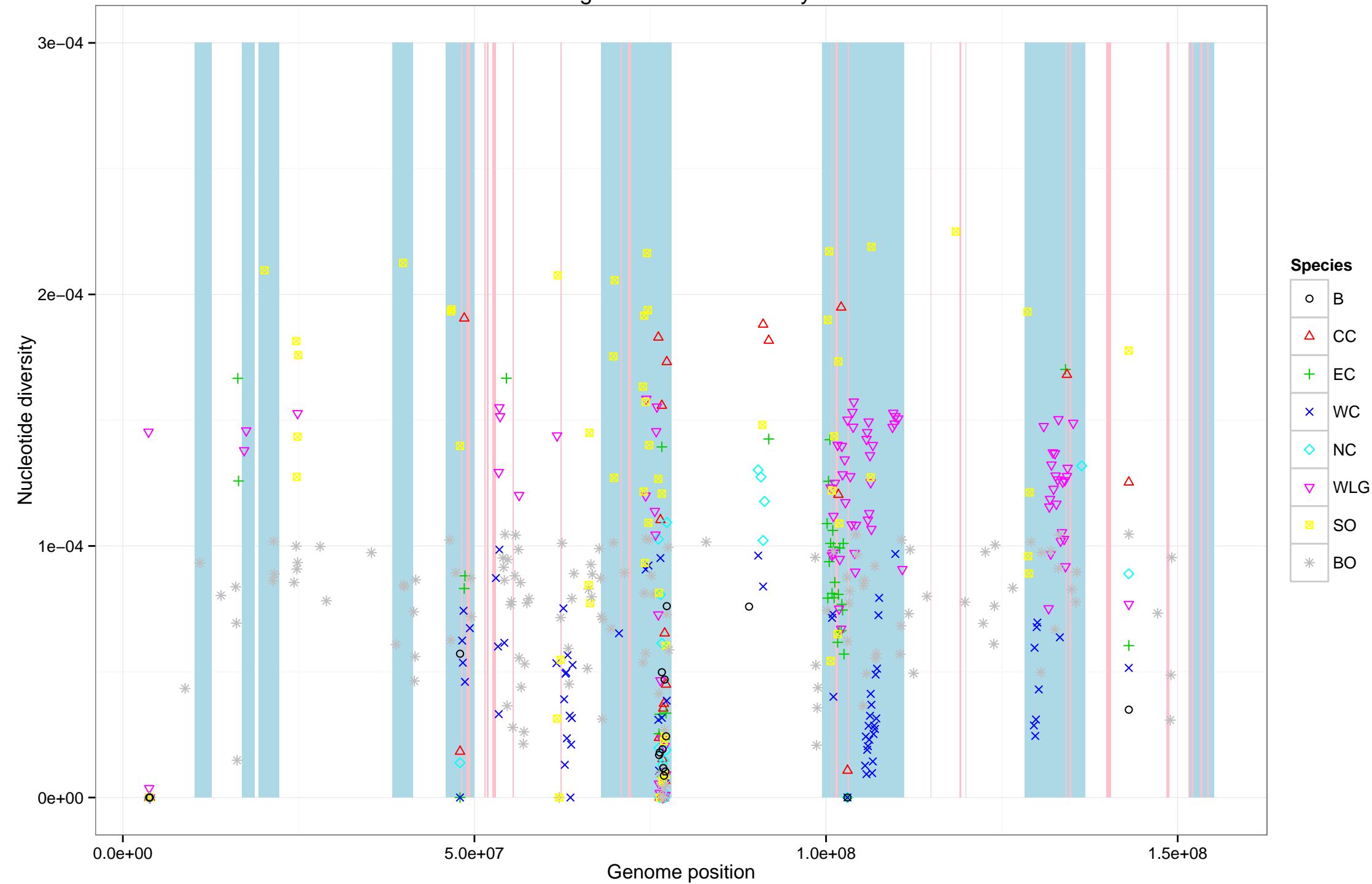

Supplement: S6 Fig — Blue bars represent low-ILS regions identified in this study. B: Bonobo, CC: Central chimpanzee, EC: Eastern chimpanzee, WC: Western chimpanzee, NC: Nigerian chimpanzee, WLG: Western lowland gorilla, SO: Sumatran orangutan, BO: Bornean orangutan. (PDF) [file pgen.1005451.s006.pdf]
